# Rule-based classifier based on accident frequency and three-stage dimensionality reduction for exploring the factors of road accident injuries

**DOI:** 10.1371/journal.pone.0272956

**Published:** 2022-08-22

**Authors:** Ching-Hsue Cheng, Jun-He Yang, Po-Chien Liu

**Affiliations:** 1 Department of Information Management, National Yunlin University of Science & Technology, Touliou, Yunlin, Taiwan; 2 Department of E-Sport Technology Management, Cheng Shiu University, Niaosong Dist., Kaohsiung City, Taiwan; University of Shanghai for Science and Technology, CHINA

## Abstract

Road accidents are one of the primary causes of death worldwide; hence, they constitute an important research field. Taiwan is a small country with a high-density population. It particularly has a considerable number of locomotives. Furthermore, Taiwan’s traffic accident fatality rate increased by 23.84% in 2019 compared with 2018, primarily because of human factors. Road safety has long been a challenging problem in Taiwanese cities. This study collected public data pertaining to traffic accidents from the Taoyuan city government in Taiwan and generated six datasets based on the various accident frequencies at the same location. To find key attributes, this study proposes a three-stage dimension reduction to filter attributes, which includes removing multicollinear attributes, the integrated attribute selection method, and statistical factor analysis. We applied five rule-based classifiers to classify six different frequency datasets and generate the rules of accident severity. The order of top ten key attributes was hit vehicle > certificate type > vehicle > action type > drive quality > escape > accident type > gender > job > trip purposes in the maximum accident frequency CF ≥ 10 dataset. When locomotives, bicycles, and people collide with other locomotives or trucks, injury or death can easily occur, and the motorcycle riders are at the highest risk. The findings of this study provide a reference for governments and stakeholders to reduce the road accident risk factors.

## 1. Introduction

According to the global road safety status report released by the World Health Organization [1], approximately 1.35 million people die in road accidents annually, which translates to an average of 3,700 daily deaths. Moreover, 50–200 million people are non-fatally injured, which often results in long-term disability. Road accidents have become the leading cause of death worldwide, and road accident injuries cause considerable economic loss to individuals, their families, and the entire country. Therefore, road safety has become a prominent issue for all countries; therefore, it is essential to prevent traffic crashes, reduce the number of casualties, and improve road safety. There are several factors that increase the risk of road traffic accidents and the resulting risk of death or injury. More than half of all road traffic injuries and deaths involve vulnerable road users such as pedestrians, cyclists, motorcyclists, and passengers. Therefore, to provide a safe traffic environment, it is crucial to understand the factors and patterns that result in traffic accidents.

Urbanization has led to an increase in the urban population, which has resulted in an increased severity of transportation problems. According to the Taiwan Ministry of Transportation and Communication [2], the number of registered cars and motorcycles has reached 22,264,293 and practically everyone owns a vehicle based on the total population ratio. According to the 2019 statistics of the Taiwan National Police Agency (NPA) [3], 341,972 traffic accidents resulted in deaths on the spot or within 24 h and injuries or more than 24 h of death. Furthermore, the number of deaths increased by 23.84% compared with that in 2018. NPA’s data [3] showed that most traffic accidents are caused because of driver errors, such as not complying with traffic rules, which accounted for the highest percentage of accidents at 19.49%; improper turning direction, which accounted for the second highest at (14.77%); violating signal control accounted for 8.05%; and 7.44% were caused by not maintaining a safe distance. Hence, most traffic accidents can be avoided or prevented in advance. Sun and Sun [4] noted that traffic accidents are closely related to traffic state and involve the responsibilities of vehicles, drivers, and roads. To prevent road accidents, the government must continue to educate drivers on traffic safety, including improving the safety performance of vehicles and understanding the risk factors for road accidents. Furthermore, they can determine the rules for traffic accidents based on historical data to formulate effective policies and regulations to avoid traffic accidents.

Several studies have explored the various factors of road traffic accidents and their impact on the risk of fatal traffic accidents [5, 6], and most used statistical methods, such as polynomial logistic [7] and logistic regression models [8]. Statistical methods offer an advantage in that they can assess the correlations between potential factors and accident severity levels, but the statistical regression model must follow certain assumptions, such as normal distribution. In contrast, rule-based machine learning (RBML) automatically improves through experience, requires fewer assumptions to use data, and fewer heuristics to generate rules for judgments and make decisions. Furthermore, rule-based methods use if-then rules to represent the nonlinear relationship between the attribute and target attribute, and understanding the discovered knowledge is easy. Therefore, to explore the factors of road accidents, this study proposes an RBML based on the frequency of accidents at a location and a three-stage dimension reduction that generates the rules of road accidents. The objectives of this study were achieved through the following:

1. Road accident data were collected from the Taoyuan city government in Taiwan, and accident frequencies at the same locations were assessed to generate different crash frequency (CF) datasets.
2. A three-stage dimension reduction method was constructed to determine the key attributes, and a simple integrated attribute selection method was proposed to synthesize the selected attributes of four classifier algorithms.
3. To generate the accident severity rules, we used five rule-based methods to classify the injury severity of road accidents and compared them with different CF datasets based on accuracy, sensitivity, specificity, area under the receiver operating characteristic curve (AUC), and F1 metrics.
4. The results of this study can be used as a reference for stakeholders to improve road safety. Furthermore, the top ten accident locations in the city were identified so that corrective measures could be implemented.

The remainder of this paper is organized as follows. Section 2 introduces related works, including road accident factors, attribute selection, and classifiers. Section 3 details the proposed method and its computational steps. Section 4 presents the experimental results and discussion, and Section 5 concludes the paper.

## 2. Related works

This section introduces the accident factors, attribute selection, and classifiers used in this study.

### 2.1 Road accident factors

Road accident data are generally divided into two types: injury severity and collision type. According to the KABCO injury classification scale [9], the severity of an injury can be fatal, incapacitating, non-incapacitating, possible, or no injury. Collision types are categorized as head-on, road departure, rear-end, side collisions, and rollovers. Factors affecting road accidents have attracted the attention of many researchers, and identifying these factors can help improve roads and educate the public, which can aid in reducing road accidents.

Road accidents are caused by a variety of factors, including road users, vehicles, road infrastructure, environment, and the interactions among these factors. In a road environment, the infrastructure design, traffic lights, and overall traffic control settings are the primary factors that affect the severity of road accidents [10]. Some studies on the factors related to road accidents, including the research variables and results, are summarized in Table 1.

**Table 1:** Studies on factors related to road accidents.

Factor	Main result	Reference
Driver, accident, vehicle, roadway, and temporal factors.	Different weather conditions have different impacts on the severity of injuries caused by truck crashes	Uddin and Huynh [11]
Victim, vehicle, road infrastructure, traffic and control, day and time, environmental factors.	Fatal accidents are more likely to occur on streets where the speed limit exceeds 40 km/h, and that males and people aged 60 years are at the most significant risk of fatal crashes.	Cantillo et al. [5]
	Motorcycles are considered to have a high probability of fatal crashes in the city.	
	There is also a high probability of fatal accidents at pedestrian bridges, traffic lights, and sidewalks.	
Accident, infrastructure, cyclists, and environmental factors	Rear-end collisions are the most dangerous type of collision.	Prati et al. [12]
	Angle collisions of trucks and cars increase the severity of injuries in cyclists.	
	Road type is a potentially important variable.	
Time, driver, and accident	Traffic flow, light conditions, road conditions, time of year, and the percentage of trucks on the road are the primary differences between time periods.	Pahukula et al. [13]
Accident, human, vehicle, road, and environmental factors.	Pedestrian accidents have an increased probability of hit-and-runs in dark driving environments, middle-aged male drivers, no driving license, and no auto insurance.	Zhang et al. [14]

Some studies have shown that age and gender are the most relevant factors related to the severity of traffic accidents [5]. Similarly, some authors have demonstrated the effects of vehicle type on accident severity [5, 12]. Furthermore, the characteristics of road infrastructure, road types, street patterns, lanes, sidewalks, and actual separation between lanes were also ascertained to be related to the severity of traffic accidents [15].

### 2.2 Attribute selection

A large amount of high-dimensional data can help in extracting valuable information, but it may present the data mining and machine learning operations with significant computational complexity and cost challenges, which is also known as dimensionality disaster. Attribute selection is a dimensionality reduction technology that can extract important attributes without reducing the data analysis performance, thereby improving the efficiency of data storage and processing [16]. It is an important preprocessing step for generating an effective machine learning model. Its purpose is to reduce the number of initial attributes and then select a subset that retains sufficient information to obtain satisfactory results. Therefore, this study identifies attributes related to road traffic accidents and deletes irrelevant or redundant attributes using correlation-based feature selection, Pearson correlation, information gain, and gain ratio. In the following section, we detail these four attribute selection methods used in this study.

#### Correlation-based feature selection (CFS)

CFS is an attribute selection method developed by Hall and Smith [17], and is a type of filtering model. It ranks attributes based on a correlation-based heuristic evaluation function [18], which evaluates a subset of attributes that are independent of each other but related to the class label. It calculates attribute-class and attribute-attribute correlations based on a good feature subset containing features that are highly related to the class. Irrelevant attributes with a low correlation to the class are ignored; hence, redundant attributes are removed because usually they are closely related to one or more other attributes.

#### Pearson correlation (PC)

PC [19] is a method used for extensive relationships between attributes, and it evaluates the strength of the relationship between two vectors based on the covariance matrix of the data. It evaluates all attributes related to a class to rank them in an order from high to low and handles the relevant attributes involved in the data exploration process. Attribute selection must first generate and rank attribute subsets. Generating attribute subsets is a search process that is used to compare candidate attribute subsets with the determined ones. If the evaluation result of the new candidate attribute subset is better than that of the previous subset, the new attribute subset is designated the important attribute set. This process is repeated until the termination condition is reached.

#### Information gain (IG)

IG [20] is widely used in high-dimensional data to measure the effectiveness of attributes in classification. It is the expected amount of information required to reduce entropy and is a filtering method that evaluates the importance of attributes by measuring the IG of related classes [21]. It calculates the information mutual to different attributes for the class, and is a very commonly used univariate method for evaluating attributes. It evaluates the importance of attributes based on the IG of a single attribute at a time and provides the entire information based on this value. In ranking attributes, a higher IG indicates a better discriminative class. It is an effective way of determining the correlations between attributes and classes, and its equation is expressed as follows:

$$IG\left(a\right)=\text{Entropy}\left(c\right)-\text{Entropy}\left(c|a\right)$$ (1)

where *c* represents class, *a* represents attribute, and p(*c* | *a*) represents the probability of *c* when *a* is known.

#### Gain ratio (GR)

The GR algorithm is an extension of the IG proposed by Quinlan [20], and it overcomes the shortcomings of IG wherein the attribute selection biases have several different values. GR standardizes IG through the split information (SI) equation, which is expressed as

$$SI_{a}\left(D\right)=-\overset{V}{\underset{i=1}{\sum }}\frac{\left|D_{v}\right|}{\left|D\right|}\times log_{2}\left(\frac{\left|D_{v}\right|}{\left|D\right|}\right)$$ (2)

where *D* denotes the training dataset and *D_v_* is the subset of partitioning *D* for attribute a. The GR can be obtained using the following formula:

$$GR_{a}\left(D\right)=\frac{IG\left(a\right)}{SI_{a}\left(D\right)}$$ (3)

When *SI*_*a*_(*D*) approaches zero, the ratio becomes unstable. To avoid this, IG of the selected test must be at least equal to the average gain of all inspection tests. Finally, the attribute with the largest GR is selected as the segmentation attribute.

### 2.3 Rule-based classifier

Several traffic accident detection models have been developed, and they can be roughly divided into statistical and machine-learning methods. Statistical methods include polynomial logistic [7], logistic regression [8], and log-linear models. Statistical methods usually contain assumptions that cannot easily handle the complex and nonlinear relationship between road traffic factors and accident risk [4]. Machine learning methods designed for nonlinear problems have recently been widely applied in traffic safety tasks. Compared with statistical models, these methods are more suitable for dealing with complex nonlinear problems. In this study, five RBML algorithms were applied to explore the road accident factors: decision tree, RIPPER, random forest, extra tree, and logistic model tree. Their descriptions are as follows:

#### Decision tree (DT)

DT is a machine learning method developed by Quinlan [20] that exhibits excellent performance in several application fields. It is a nonlinear and non-parametric data-mining tool that can be used for supervised classification and in regression problems. It is easy to use, explains mathematics simply without complicated equations, and visually presents all analysis results in an easy-to-understand hierarchical tree diagram (or if-then) with only a brief description for quick comparison. In machine learning, DT is widely used in traffic safety studies, and it can quickly identify and easily interpret complex patterns related to road crashes [22]. To overcome the shortcomings of statistical methods, DT can be selected as the first method to identify road accidents and allow for an easy understanding of the results. Unlike statistical methods, DT does not require any prior assumptions about or restrictions on the relationship between attributes and it can overcome the multicollinearity problem.

#### Repeated incremental pruning to produce error reduction (RIPPER)

RIPPER is a rule-based classifier proposed by Cohen [23]. It is a widely used rule induction algorithm that derives a set of rules from the training set. The RIPPER algorithm can be divided into three steps: growth, pruning, and optimization. In the first step, the divide and conquer method is used to add conditions to the rule until it is perfectly classified as a subset of the data. Similar to a DT, the IG criterion is used to identify the next split attribute. In the second step, when the specificity of the rule is increased, entropy is no longer reduced, and the rule is immediately pruned. In the third step, the first and second steps are repeated until the stopping criterion is reached. Various heuristics are used to optimize all the rules. The advantage of RIPPER is that the rules are relatively easy to interpret and suitable for unbalanced problems [24].

#### Random forest (RF)

The RF algorithm combines the concept of the random subspace method [25] and bootstrap aggregation, which was developed by Breiman [26]. RF is a process of creating several different DTs with different samples at each node, and using the score of each DT as the average of its final scores to obtain more accurate results. For outliers and imbalanced datasets, RF is more robust and scalable than DTs and can overcome nonlinear trends. Furthermore, it reduces bias and overfitting by using multiple trees to reorganize the training data. It is worth noting that RF’s success in providing high-precision prediction is primarily achieved by solving the trade-off between overfitting and prediction accuracy [26]. It can provide high and stable classification performance, the workload of adjusting its parameters is small, and it can be applied to classification and regression cases. Compared with multiple regression and neural networks, RF is highly interpretable and does not require any specific data distribution and normalization of variables in different ranges because it does not require rescaling, transformation, or modification [27].

#### Extra tree (ET)

ET is a machine learning algorithm that is an extension of the RF algorithm proposed by Geurts et al. [28]. It increases the randomness of the RF algorithm, and its operation is very similar to that of RF. However, the method of building DT in forests is different. There are two main differences between ET and RF. First, RF employs random sampling (bootstrapping) to select the sampling dataset as the training dataset of each DT, whereas ET generally does not employ random sampling; therefore, each DT uses the original training dataset. Second, RF chooses the best split point to partition the DT, whereas ET selects the split point randomly. Once the split point is selected, these two algorithms select the best subset among all the attribute subsets. Therefore, ET adds randomization and still retains optimization. These two differences prevent ET from overfitting and enables it to provide a better performance [28].

#### Logistic model tree (LMT)

LMT is a classifier proposed by Landwehr et al. [29] that combines DT and linear logistic regression. It uses a LogitBoost algorithm [30] to gradually improve the logistic regression model along the corresponding path from the root to the leaf. In the LMT operation, each intermediate node is split based on the gain ratio, and the LogitBoost algorithm is used to update the node model inherited from the parent node. Furthermore, the LogitBoost algorithm generates a linear regression model for each node in the tree, and the LMT uses the CART algorithm [31] to prune the tree. This learning method can overcome the problems associated with local learning. Furthermore, LMT uses simple logistic variable selection to reduce the number of parameters in the logistic regression and improve the classification performance. It uses cross-validation to find multiple LogitBoost iterations to prevent overfitting of the training data, and the LogitBoost algorithm uses additive logistic regression with least-squares fitting for each class.

## 3. Proposed method

According to a global road safety report issued by the World Health Organization [1], approximately 1.35 million people die in road traffic accidents each year. This high number of deaths can be considered to be because of a lack of awareness of the risk factors that may result in traffic accidents or possible injuries. Therefore, research on traffic accidents is required to prevent these casualties because most road accidents are caused by human factors. Several analytical methods have been used to study the various potential factors that can cause traffic accidents. According to Chand et al. [32], previous analysis methods for road accidents can be described as follows: Building and checking classification rules related to traffic accident data; selecting the significant factors that caused an accident to accurately model the road accident; developing driver’s rules and behavior on the road; selecting important features for training deep learning algorithms and artificial neural networks; and distinguishing between safe and unsafe driving areas. From these five methods, we can roughly summarize these previous research methods as statistical and machine learning methods.

The most common statistical methods are parametric regression models, such as multinomial logit regression, ordered probit, and logit regression, which are applied to explore correlations. Statistical models rely on parameter estimates, and they have their own model assumptions and predefined basic relationships between dependent and independent variables. If these assumptions are violated, the model may result in erroneous estimates of accident probability [7], which may also hinder the discovery of complex associations between crucial factors. However, machine learning involves iterative learning of structures, rules, and hidden patterns based on a large amount of data. It is not limited by statistical assumptions. Furthermore, it enables discovery of valuable relationships that have not been discovered by existing research.

The traditional view is that the rule sets of machine learning and data mining are more interpretable than those of other models, and that these rule-based models are simple models that are more interpretable than the more complex ones [33]. RBML uses human experience and fewer assumptions to train the data, and heuristics to generate rules. Furthermore, the rule-based methods use if-then rules to represent the relationship between attributes and the target class, and the analysis results are easy to understand. The RBML has the following advantages [34]: its rules allow the machine to implement the best strategy for its environment. These rules do not contain unnecessary information; therefore, a minimal set of rules is created. Furthermore, the rule set generated through RBML is a double-optimization problem.

In this study, five RBML algorithms that were applied to explore factors causing road accidents are DT, RIPPER, RF, ET, and LMT, which were selected for the following reasons:

- The five classifiers must be rule-based, and the computational cost and expenses of a rule-based classifier is less than that of neural networks and deep learning.
- The DT is a commonly used baseline method, and DT and RIPPER are easy to implement and contain no assumptions. Their tree generates decision rules that are easy to understand for the users.
- RF and ET are rule-based ensemble classifiers (RBECs). RBECs generally perform better than the individual classifiers that construct them and overcome the limitations of the individual classifiers. Furthermore, ET is an extension of the RF.
- The LMT combines DT and linear logistic regression, and uses simple logistic variable selection to reduce the number of parameters in the logistic regression and improve the classification performance.

Based on the above, previous research has room for improvement, and RBML algorithms have several advantages. Furthermore, most previous studies were based on vehicle type (bicycle, bus, and motorcycle), vulnerable road users, and collision type (single vehicle, multiple vehicles, and lane departure accident) for describing the causal factors of road traffic accidents. However, few studies have been conducted on the frequency of recurring accidents at the same location. The purpose of this study was to investigate the potential factors of recurring accidents at a location that result in the degree of injury in traffic accidents, and to generate their rules. Therefore, this study proposes RBML based on the frequency of accidents at a location, and three-stage dimension reduction to explore the attributes of the accident severity level. Three-stage dimension reduction includes removal of multicollinear attributes, the integrated attribute selection method (COM_3), and statistical factor analysis. The three-stage dimension reduction was proposed because:

- Statistical methods outperform other models in assessing the correlations between potential factors and accident severity levels [32]. Therefore, we used a multicollinear regression diagnosis to delete the attributes with collinearity problems.
- The machine learning attribute selection method was used in the second stage because it can handle the nonlinear relationship between the attributes and target class, and important attributes can be selected based on the degree of correlation with the target class.
- The third stage uses factor analysis (FA) because it can be used to examine whether the identified attributes lack cohesion and delete the attributes with insufficient cohesion. It can rename the factor dimension to determine the clustering status and intensity of the attributes.

### 3.1 Proposed computational step

**Fig 1:**
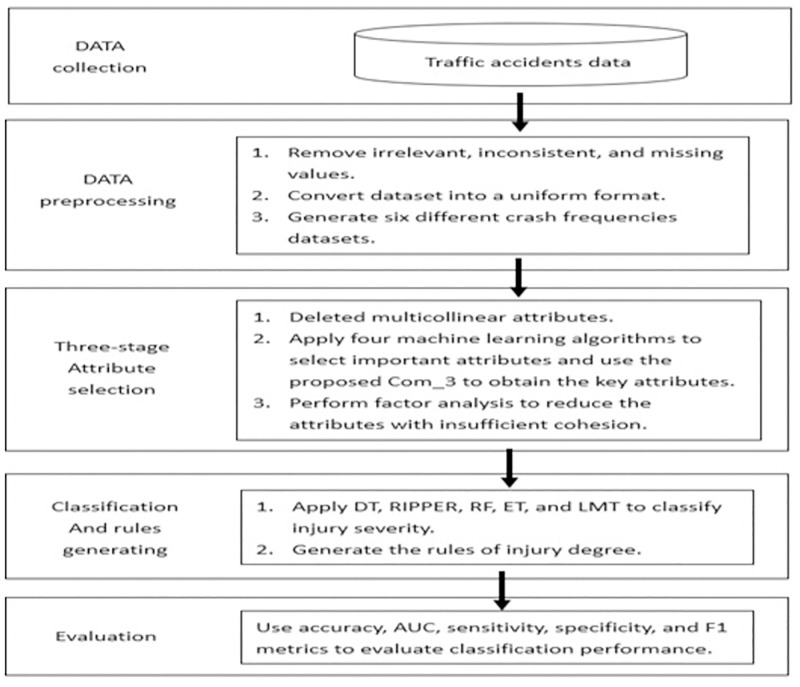
Proposed computational steps.

To simplify the understanding of the proposed method, we divided the entire research process into five computational steps, as shown in Fig 1. These steps include data collection, pre-processing, attribute selection, classification (generation rules), and evaluation. A detailed description of each step is provided below:

#### Step 1: Data collection

This data used in this study was obtained in Taoyuan City, which is one of the six largest cities in Taiwan. It has an area of 1,220 square kilometers and population of 2,271,969. Compared with other counties and cities in Taiwan, wherein the average population growth rate is decreasing, Taoyuan City continues to show a growth trend. Owing to the city’s large population, traffic flow is significant. During peak hours or holidays, numerous people and vehicles flow in urban areas or between cities, which causes frequent traffic jams on several important roads in urban areas, highways, and expressways. Compared with other metropolitan cities in Taiwan, Taoyuan City has the highest growth rate in the number of motor vehicle registrations because its population has continued to increase in recent years.

This study obtained public data on road traffic accidents from the Taoyuan City government. The data contained the road environment characteristics and related factors of the accidents that occurred between 2017–2018, and comprised 123,365 records with 109 attributes. The collected road accident cases were carefully recorded by the policies in semi-structured texts and numbers. These attributes included demographics related to drivers (age, gender, driver license, drinking status, degree of injury), road (light, road surface, sight distance, traffic signs), environment (accident location, time, date, weather), and vehicle dimensions (vehicle type, purpose of vehicle use, areas of the vehicle involved in the collision).

#### Step 2: Data preprocessing

Data mining is a step in the knowledge discovery in databases process, and its primary objective is to extract high-level knowledge from low-level information. That is, handling a large amount of raw data automatically is necessary to identify the most important and meaningful patterns. Each data-mining process requires a data preprocessing step [35], and its main purposes are to remove irrelevant, inconsistent, and missing values in a dataset, convert the dataset into a uniform format, and sort and induce the dataset for further use. The data preprocessing used in this study is described as follows:

1. Remove irrelevant, inconsistent, and missing values: To delete useless data, irrelevant and inconsistent attributes were removed, and then the attributes with more than half of the missing values were deleted. Furthermore, observations with missing values were also deleted. After removing the attributes and data, the collected data retained 39 attributes (including classes) with 83,875 records.
2. Convert the dataset into a uniform format: In the original data, some attributes were found to be similar; hence, we merged these attributes into a single attribute. Furthermore, we renamed the attributes with inconsistent names and reorganized all columns and rows in a uniform format.
3. Code semi-structured data into a structured format: In this step, we coded some attributes into binary zero-one (no or yes) representations, such as sight distance quality, pavement edge line, drunkenness, and escaping the accident. To simplify attribute values, if a nominal/ordinal attribute had too many linguistic values, we merged some linguistic values into a single linguistic value. For example, traffic roads included national, provincial, county, country, urban, village, dedicated, and agricultural production roads; we merged agricultural production roads with few occurrences into a reasonably similar village road. In the class attribute, there were four accident severity levels: (1) dead within 24 h, (2) dead within 2–30 days, (3) injured, and (4) uninjured. Given that the samples of some accident severity levels were small, a class imbalance could occur. Therefore, we merged accident severity levels (1), (2), and (3) into class Y (injury or death) and represented uninjured as class N (uninjured). The attribute definitions and values of the complete processed data are listed in Table 2.
4. Generate different CF datasets: After the collected data were processed through steps (1), (2), and (3), we wrote a Python program to search for the number of accident occurrences in the same location from the district, road section (street), lane, number, and road intersection of the accident location. Eventually, we generated six datasets with accident frequencies greater than 2, 3, 4, 5, 8, and 10, and they were named CF ≥ 2, CF ≥ 3, CF ≥ 4, CF ≥ 5, CF ≥ 8, and CF ≥ 10, respectively.

**Table 2:** Attribute definitions and collected data values.

Attribute	Abbr.	Description	Values
Light condition	Light	Light condition	Daylight, twilight, illuminated at night, no lighting at night
Road class	Road_c	Administrative classification of roads	Provincial, county, township, urban, village, dedicated road, and other
Over speed	Speed_o	Exceeding the speed limit	Yes or no
Road type	Road_t	Road type	Railroad crossing, intersection road, straight road, traffic circle (roundabout)
Accident location	Accid_l	Accident location	Intersection, straight road, highway interchanges, and other
Road surface	Road_su	Road surface pavement	Asphalt, cement, gravel, other paving, and no paving
Road condition	Road_co	Condition of the road surface	Snow, slick, muddy, wet, and dry
Road defect	Road_de	Road surface defect	Soft terrain, prominent unevenness, potholes, no defects
Obstacle	Obstacle	Obstacles on the road	Road under maintenance, piled objects, parking on the road, other obstacles, and no obstacles
Sight distance quality	Sight_q	The quality of distance visible to the driver of a vehicle	Bad or good
Sight distance	Sight	The distance visible to the driver of a vehicle	Curve road, ramp road, buildings, roadside trees, crops and vehicles, good, and other
Signal type	Sign_ty	Traffic signal type	Traffic control, multi-function traffic control, flashing signal, and no setting
Signal status	Sign_st	Traffic control signal status	Normal, abnormal, no signal setting
Direction restriction	Direct	Directional restriction setting	Divisional island, two-way no overtaking, one-way no overtaking, overtaking permitted, no setting
Separating fast and general lanes	Sep_FG	Separating fast (passing) and general (express) lanes	Forbidding lane changing with a sign, forbidding lane changing with no sign, lane line with a sign, lane line with no sign, and no lane line
Separating fast and slow lanes	Sep_FS	Separating fast (passing) and slow (local) lanes	Wide fast and slow lanes separation (above 50 cm), narrow fast and slow lanes separation (with fence), narrow fast and slow lanes separation (no fence), a line separating fast and slow lanes, no fast and slow lane separation.
Pavement edge line	Edge	pavement edge line	Yes or no
Time	Time	Time of accident occurrence	Morning (6:00–12:00), afternoon (12:00–18:00), and evening (18:00–6:00)
Month	Month	The month of accident occurrence	January, February, March. April, May, June, July, August, September, October, November, and December
Week	Week	Week of accident occurrence	Monday, Tuesday, Wednesday, Thursday, Friday, Saturday, and Sunday
District	District	District of accident occurrence	Bade, Daxi, Dayuan, Guanyin, Guishan, Longtan, Luzhu, Pingzhen, Taoyuan, Xinwu, Yangmei, Zhongli, and Fuxing
Weather	Weather	The weather of accident occurrence	Rain, strong wind, fog or smoke, overcast, and sunny
Vehicle type	Vehicle	Vehicle type of driver	Passenger cars, trucks, motorcycles, and others
Vehicle purpose	Veh-p	Purpose of using the vehicle	Passengers, goods, and others
Hit vehicle	Hit_veh	Vehicle type of victim	Automobiles, motorcycles, and others
Gender	Gender	Gender of victim	Male or female
Age	Age	Age of victim	< 18, 18–23, 24–39, 40–64, and > 64 years
Protective equipment	Pro_eq	Protective equipment of victim	Wearing a safety helmet or belt, not wearing a safety helmet or belt, others (pedestrians, bicycles, etc.)
Electronic devices use	E-use	Using mobile phones or related electronic devices while driving	Not using, using mobile phones/electronic devices and hindering driving safety, using hands-free mobile phones/electronic devices without hindering driving safety, non-drivers using mobile phones/electronic devices and hindering driving safety
Driving license	Driver_q	Certificate of driver	Yes or no
Certificate type	Certifi_t	Types of driver’s license	Professional, general, motorcycle, military driver’s license, and others
Drunk	Druck	The driver consumed alcohol	Yes or no
Escaping the accident	Escape	Driver escapes the accident	Yes or no
Job	Job	Occupation of driver	Public opinion representatives and supervisors (managers), professionals, technicians and assistant professionals, business support staff, service and sales staff, production staff (agricultural, forestry, fishing, and husbandry), housewives/husbands, machinery and equipment operators, non-skilled and manual workers, others
Itinerary purpose	Trip_p	Itinerary purpose of driver	Commute to work, commute to school, business contacts, transportation, social activities, sightseeing tours, shopping, and others
Action type	Action_t	Type of taking action to respond the moment of collision	Vehicle or human action
Accident type	Accident_t	Accident type of accident	People and vehicle, vehicle and vehicle, and only the vehicle
Accident cause	Accident_c	The cause of the collision	Drivers, lights, loading, parts, pedestrians/passengers, traffic control facilities, none (non-vehicle driver factors), and others
Severity degree	Severity	Injury severity degree	class Y (injury or death with 51,098 records) and class N (uninjured with 32,777 records)

#### Step 3: Three-stage dimension reduction

Attribute selection is a dimensionality reduction technique that solves the problem of determining the most useful attribute set for a given problem and improves the efficiency of data storage and processing [36]. The following section details the proposed three-stage dimension-reduction method.

- Stage 1: Removing multicollinear attributes. This stage applies the variance inflation factor (VIF) to diagnose the collinearity problem through multiple linear regression [37]. The VIF is the ratio of the variances of the cases when there is multicollinearity between the interpretation variables and when there is no multicollinearity. According to Hair et al. [37], when VIF = 1 is not correlated, 1 < VIF < 5 is moderately correlated and VIF ≥ 5 is highly correlated. Furthermore, if VIF ≥ 4, we need to examine whether there is a multicollinearity problem. Therefore, this stage deletes the independent variable when its VIF value is greater than or equal to 4 (VIF ≥ 4).
- Stage 2: Machine learning algorithm to select attributes. This stage first proposes a simple integrated attribute selection method wherein the key attributes must appear more than three times in the four attribute selection methods; it is called COM_3. This stage uses the CFS, PC, IG, and GR attribute selection methods to identify the important attributes that affect accident severity. We then apply the proposed COM_3 to integrate the important attributes of the four attribute selection methods to obtain the key attributes.
- Stage 3: Factor analysis (FA) to reduce data dimension. FA is a generalization of principal component analysis that reduces numerous attributes to fewer dimensions (factors). The primary objective of FA is to transform the coordinate system in order to minimize the correlation between the system variables [38]. There are several methods to determine the number of dimensions extracted through FA, and the most widely used method is the principal component with eigenvalues greater than one as the dimensions [39]. Furthermore, we used the FA results to delete attributes with low loadings. As a rule of thumb, we recommend interpreting only factor loadings with an absolute value greater than 0.5, which explains approximately 25% of the variance [37].
- Step 4: Classification and rule generation. To explore the relationship between the attributes and injury degree, this step uses five RBML classifiers to classify six different accident frequency datasets and generate their rule sets. The five RBML classifiers are DT, RIPPER, RF, ET, and LMT, and they were selected for the following reasons: (1) DT is a commonly used baseline method, and DT and RIPPER are easy to implement. (2) RF and ET are ensemble classifiers, and ET is an extension of the RF. (3) LMT combines DT and linear logistic regression. In the experiment, each dataset was implemented using 10-fold cross-validation, and the average of 100 repeated implementations was calculated to present their results for comparing their performance. Finally, the rule sets were generated in an IF-THEN form, and a DT diagram was used to easily understand the key attributes and study results.
- Step 5: Evaluation. Evaluation is a standard method for measuring the effectiveness of an RBML classifier, which uses accuracy, sensitivity, specificity, AUC, and F1 metrics to evaluate the performance of the five RBML classifiers. This study used AUC because the receiver operating characteristic curve (ROC) has diagnostic power in imbalanced classes, and it can handle both the positive detection and false alarm rates. Furthermore, AUC is a measure of the discriminative strength between these two rates without considering misclassification costs or class prior probabilities [40]. F1 (F-score or F-measure) is a widely used measurement standard in information retrieval and class imbalance problems, and it is the harmonic mean between precision and sensitivity [41]. The classification results were calculated using the confusion matrix [42], which has two dimensions. One is the actual class of the object and the other is the class predicted by the classifier. The accuracy, sensitivity, specificity, and F1 metrics are based on the confusion matrix, and their equations are expressed as follows [41]:
$$Accuracy=\frac{TP+TN}{TP+TN+FP+FN}\times 100$$ (4)
$$Sensitivity=\frac{TP}{TP+FN}$$ (5)
$$Specificity=\frac{TN}{TN+FP}$$ (6)
$$F1=\;\frac{2TP}{2TP+FP+FN}$$ (7)
- where TP, TN, FP, and FN represent true positive, true negative, false positive, and false negative, respectively.

## 4. Results and discussions

This section presents the datasets and parameter settings used, and experimental results obtained in this study. It also presents a discussion of the results.

### 4.1 Experimental datasets and parameter settings

This study collected public data on road traffic accidents from the police department of Taoyuan City in Taiwan. The traffic accident data was from the 2017 to 2018 period and contained 123,365 records with 109 attributes. The police recorded the accident cases in semi-structured text and numbers, and their attributes included demographics related to drivers, road, environment, and vehicle dimensions. After the computational step 2 of data preprocessing, 83,875 records of 39 attributes (including one class attribute) were retained as complete data, and their attribute definitions and values are listed in Table 2. In this study, the target class was the severity of the road accident, that is, the degree of injury caused by traffic accidents to road users. The original target class had four accident severity levels because of the small number of records in some classes, which caused class imbalance. Therefore, we merged the three classes into class Y (injury or death) and retained class N (uninjured). Next, we coded a Python program to screen the number of accident occurrences in the same location from the district, road section (street), lane, number, and road intersection of the occurrence location. Finally, we generated six datasets with different accident frequencies and named them CF ≥ 2, CF ≥ 3, CF ≥ 4, CF ≥ 5, CF ≥ 8, and CF ≥ 10. The dataset records, class records, and class ratios are listed in Table 3, which indicates that the seven datasets had a slightly imbalanced class.

**Table 3:** Dataset records, class records, and class ratios in the experimental datasets.

Frequencies	Dataset records	Class records (Y: N)	Class ratios
Complete data	83875	51098: 32777	1.56
CF ≥ 2	39397	23948: 15449	1.55
CF ≥ 3	19156	11519: 7637	1.51
CF ≥ 4	19156	11519: 7637	1.51
CF ≥ 5	12511	7491: 5020	1.49
CF ≥ 8	9212	5471: 3741	1.46
CF ≥ 10	7108	4208: 2900	1.45

To process and visualize data, the experimental environment comprised Python 3.7.4 running on a computer with a 3.6 GHz Intel i7-7700 CPU and Windows 10 operating system. The five RBML classifiers were applied to classify the datasets and generate their rule sets; their parameter settings are listed in Table 4.

**Table 4:** Parameter settings of the five RBML classifiers.

Classifier	Parameter	Reference
DT	Confidence factor: 0.25	Quinlan [20]
	Minimum number of instances: 3	
	Folds: 3	
RIPPER	Folds: 3	Cohen [23]
	Minimal weights: 2.0	
RF	Iterations: 100	Breiman [26]
	Batch-size: 100	
ET	Iterations: 10	Geurts et al. [28]
LMT	Boosting iterations: 2	Landwehr et al. [29]

### 4.2 Experimental results

This section illustrates data visualization, finding key attributes through three-stage dimension reduction, classification (generating rules), and a comparison of the proposed method.

#### 4.2.1 Data visualization

To visualize the data, we used the Geocoding API of Google Maps to obtain the latitude and longitude based on the location (address) of the road accidents and then applied the Choropleth and HeatMap map visualizations in the Python tool to generate visual maps. The dataset shows that the Taoyuan, Guishan, and Luzhu districts had more traffic accidents than other districts. The Fuxing district is a mountainous area of aboriginal people with few accident accidents. We also screened the top ten traffic accident locations in Table 5.

**Table 5:** Top 10 traffic accident locations in Touyuan City.

District	Road intersection or address	Location characteristics	Frq.
Guishan	Intersection of Wenhua 1st Road and Guishan 1st Road	Large-scale hospitals and industrial areas	68
Bade	Intersection of Jieshou Road, Section 2 and Heping Road	Densely populated dining area and hypermarket	68
Taoyuan	Intersection of Daxing West Road, Section 3 and Zhengguang Road	Important location for court and highway interchange	54
Pingzhen	Intersection of Zhongfeng Road and Yanping Road	Dining area and green park	50
Pingzhen	Intersection of Huannan Road and Fudan Road	Hospital	46
Zhongli	Intersection of Xinzhong North Road and Puzhong Road	An important location for students of Chung Yuan Christian University	46
Zhongli	Intersection of Huanzhong East Road and Puzhong Road	An important location for students of Chung Yuan Christian University	46
Bade	176 Zhonghua Road	Hospitals and hypermarkets	42
Taoyuan	Intersection of Zhongzheng Road and Ciwen Road	Densely populated important location	38
Taoyuan	Intersection of Jieshou Road and Changsha Street	Important dining area and green park	38

#### 4.2.2 Finding key attributes through three-stage dimension reduction

Through Step 3 of the proposed computational steps, the experiment used the proposed three-stage dimension reduction to sequentially determine the key attributes. Here, we illustrate only the CF ≥10 dataset to determine the key attributes because this dataset has important management implications for exploring the key attributes of road accidents.

- The VIF is used to delete collinear attributes. The dataset contained 38 key attributes and one class after COM_3 integrated attribute selection. Next, we used multiple linear regression [37] to generate the VIF for diagnosing the collinearity problem, and used a VIF value greater than or equal to four [37] to delete the collinear attributes, as illustrated in Table 6. We observed that VIF ≥ 4 for four attributes (Sign_ty, Sign_st, Pro_eq, and E_use). Thereafter, we deleted these four attributes before the next stage of the analysis.
- COM_3 was used to integrate the important attributes of the four attribute selection methods. After the FA deleted four attributes, the dataset retained 34 conditional attributes and one class attribute. This study applied CFS, PC, IG, and GR to identify the important attributes, and then used the proposed COM_3 to integrate these important attributes from the four attribute selection methods. We only list the results of attributes selected by at least three attribute selection methods in Table 7, and these 11 attributes are the key attributes.
- Factor analysis (FA) was applied to reduce the attributes. FA was used to obtain the five principal components with eigenvalues greater than or equal to one (λ ≥ 1, the total variance explained 72.48%), as listed in Table 8, and deleted the age attributes with an absolute value of loading less than 0.5 [37], as listed in Table 8. Therefore, we renamed the five principal components to vehicle-related (vehicle), experience and skill, work-related (work), avoid responsibility, and gender dimensions.

**Table 6:** Results of the collinearity test using VIF of multiple linear regression.

Attribute	Standardized β	t statistics	significance	VIF	Attribute	Standardized β	t statistics	significance	VIF
Time	-0.030	-3.666	0.000	1.276	Sep_FG	-0.003	-0.315	0.753	1.585
Month	-0.005	-0.661	0.509	1.028	Sep_FS	0.007	0.858	0.391	1.260
Week	-0.001	-0.137	0.891	1.015	Edge	-0.003	-0.321	0.748	1.773
District	0.000	0.050	0.960	1.218	Accident_t	0.083	9.839	0.000	1.316
Weather	0.022	1.585	0.113	3.556	Accident_c	-0.017	-2.272	0.023	1.071
Light	0.013	1.586	0.113	1.328	Gender	0.045	6.046	0.000	1.057
Speed_o	0.013	1.754	0.079	1.037	Age	-0.004	-0.487	0.626	1.137
Road_c	0.031	4.211	0.000	1.030	Vehicle	0.546	39.709	0.000	3.534
Speed_o	0.002	0.141	0.888	3.065	Pro_eq	-0.068	-4.025	0.000	**5.260**
Road_t	0.012	0.975	0.330	2.792	E_use	-0.002	-0.114	0.909	**4.607**
Accid_l	0.001	0.099	0.921	1.009	Veh_p	-0.009	-1.240	0.215	1.022
Road_su	-0.017	-1.241	0.214	3.559	Action_t	0.016	1.482	0.138	2.123
Road_co	0.013	1.820	0.069	1.017	Driver_q	-0.157	-12.048	0.000	3.159
Road_de	0.000	-0.059	0.953	1.028	Certifi_t	0.193	15.193	0.000	3.026
Sight_q	-0.011	-1.359	0.174	1.132	Drunk	0.021	2.858	0.004	1.036
Sight	0.000	-0.038	0.969	1.100	Hit_veh	0.164	13.274	0.000	2.833
Sign_ty	0.024	1.516	0.130	**4.708**	Escape	-0.035	-4.686	0.000	1.021
Sign_st	-0.018	-1.138	0.255	**4.926**	Job	0.012	1.452	0.147	1.357
Direct	-0.007	-0.744	0.457	1.659	Trip_p	-0.017	-2.077	0.038	1.265

Note: The bold numbers denote VIF > 4, and these attributes are deleted in the next stage.

**Table 7:** Results of the four attribute selection methods and COM_3 in the CF ≥ 10 dataset.

Attribute	CFS	PC	GR	IG	Com_3
Accident_t		V	V	V	V
Gender		V	V	V	V
Age		V	V	V	V
Vehicle	V	V	V	V	V
Action_t		V	V	V	V
Driver_q		V	V	V	V
Certifi_t	V	V	V	V	V
Hit_veh	V	V	V	V	V
Escape	V	V	V	V	V
Job		V	V	V	V
Trip_p		V	V	V	V

**Table 8:** Results of factor analysis in CF ≥ 10 dataset.

Attribute	Vehicle	Experience and skill	Work	Gender	Avoid responsibility
Accident_t		-0.739			
Gender				0.877	
Age					
Vehicle	0.903				
Action_t		0.808			
Driver_q		0.656			
Certifi_t	0.852				
Hit_veh	0.870				
Escape					0.975
Job			0.834		
Trip_p			0.836		

Note: The blank spaces denote that the absolute value of the loading was less than 0.5.

#### 4.2.3 Classification (generating rules) and comparison

From the proposed computational Steps 4 and 5 in Section 3, we used the five RBML classifiers (DT, RIPPER, RF, ET, and LMT) to classify the injury severity and generate rules, and then compared their performance based on five metrics. The experiment applied tenfold cross-validation and the average of 100 repeat implementations to obtain their results. From Table 3, it is evident that the seven datasets have a slight class imbalance problem; hence, we used the accuracy, sensitivity, specificity, AUC, and F1 metrics to evaluate the performance. The following describes the performance of the five RBML classifiers in the datasets of different accident frequencies, performance of the three-stage dimension reduction in the most valuable complete datasets CR ≥ 1 and CR ≥ 10, and the generation of DT rules.

- Comparing different accident frequency datasets. The results of six datasets with different accident frequencies are listed in Table 9. CR ≥ 3 and CR ≥ 4 had the same records and metric results; therefore, we only list the results of CR ≥ 4. The results indicate that LMT is the best RBML classifier for the five metrics, except for the sensitivity of the CR ≥ 5 dataset. Furthermore, the best accuracy obtained was 91.96% in the CR ≥ 4 dataset, and the highest AUC of the six datasets was 0.93, except for the CR ≥ 10 dataset. The best sensitivity was 0.97 for the complete dataset (CR ≥ 1), and the highest specificity of the six datasets was 0.97, except for the CR ≥ 1 dataset. The highest F1 in the complete dataset (CR ≥ 1) was 0.93. In summary, LMT is the best RBML classifier for the six different accident frequency datasets.
- Comparing different stage dimension reductions. This study illustrates the performance of each stage dimension reduction in the most valuable complete datasets CR ≥ 1 and CR ≥ 10; the results are listed in Table 10. In the CR ≥ 1 dataset, the best performance was that of the LMT classifiers in the five metrics. Furthermore, we observed the following: (i) after removing collinear attributes, the accuracy of the C4.5, JRip, and RF classifiers improved; (ii) the most important task was to remove collinear attributes and use COM_3 to integrate the key attributes, which also improved the accuracy of the C4.5, Jrip, and LMT classifiers; (iii) we applied FA to delete the attributes with insufficient cohesion, and although all performances did not improve further, the results of FA renamed the principal components.

**Table 9:** Results of the six datasets (full attributes) based on different accident frequencies.

Dataset	Metric	DT	RIPPER	RF	ET	LMT
Complete dataset CR ≥ 1 (83875)	Accuracy	91.61	91.74	91.54	90.91	**91.78**
	AUC	0.91	0.90	**0.93**	0.92	**0.93**
	Sensitivity	0.96	**0.97**	0.96	0.96	**0.97**
	Specificity	**0.84**	**0.84**	**0.84**	**0.84**	**0.84**
	F-measure	**0.93**	**0.93**	**0.93**	**0.93**	**0.93**
CR ≥2 (39397)	Accuracy	91.39	91.55	91.33	90.14	**91.65**
	AUC	0.91	0.90	0.92	0.91	**0.93**
	Sensitivity	**0.84**	**0.84**	**0.84**	**0.84**	**0.84**
	Specificity	0.96	**0.97**	0.96	0.94	**0.97**
	F-measure	0.88	**0.89**	0.88	0.87	**0.89**
CR ≥4 (19156)	Accuracy	91.72	91.80	91.49	90.22	**91.96**
	AUC	0.91	0.91	0.92	0.91	**0.93**
	Sensitivity	**0.85**	0.84	0.84	**0.85**	**0.85**
	Specificity	0.96	**0.97**	0.96	0.94	**0.97**
	F-measure	**0.89**	**0.89**	**0.89**	0.87	**0.89**
CR ≥5 (12511)	Accuracy	91.64	91.54	91.47	90.17	**91.88**
	AUC	0.91	0.90	**0.93**	0.92	**0.93**
	Sensitivity	**0.85**	0.84	0.84	0.84	0.84
	Specificity	0.96	**0.97**	0.96	0.94	**0.97**
	F-measure	**0.89**	**0.89**	**0.89**	0.87	**0.89**
CR ≥8 (9212)	Accuracy	91.31	91.37	91.25	89.82	**91.68**
	AUC	0.90	0.90	0.92	0.91	**0.93**
	Sensitivity	**0.84**	**0.84**	**0.84**	**0.84**	**0.84**
	Specificity	0.96	0.96	0.96	0.94	**0.97**
	F-measure	**0.89**	**0.89**	**0.89**	0.87	**0.89**
CR ≥10 (7108)	Accuracy	91.44	91.45	91.35	89.90	91.66
	AUC	0.90	0.91	**0.92**	**0.92**	**0.92**
	Sensitivity	0.84	**0.85**	**0.85**	**0.85**	**0.85**
	Specificity	**0.96**	**0.96**	**0.96**	0.93	**0.96**
	F-measure	**0.89**	**0.89**	**0.89**	0.87	**0.89**

Note: Bold numbers denote the best performance of each metric among the five RBML classifiers.

**Table 10:** Results of three-stage dimension reduction for CF ≥ 1 and CF ≥ 10 datasets.

Dataset	Metric	C4.5	JRip	RF	Extra Tree	LMT
CF ≥ 1 (full attributes)	Accuracy	91.61	91.74	91.54	90.91	91.78
	AUC	0.91	0.90	0.93	0.92	0.93
	Sensitivity	0.96	0.97	0.96	0.96	0.97
	Specificity	0.84	0.84	0.84	0.84	0.84
	F-measure	0.93	0.93	0.93	0.93	0.93
CR ≥ 1 (removing collinearity)	Accuracy	**91.62**	**91.75**	**91.56**	90.91	91.78
	AUC	0.91	0.90	0.93	0.92	0.93
	Sensitivity	0.96	0.97	0.96	0.96	0.97
	Specificity	0.84	0.84	0.84	0.84	0.84
	F-measure	0.93	0.93	0.93	0.93	0.93
CR ≥ 1 (removing collinearity + COM_3)	Accuracy	**91.72**	**91.75**	91.27	90.18	**91.80**
	AUC	0.91	0.90	0.92	0.91	0.93
	Sensitivity	0.96	0.97	0.96	0.94	0.97
	Specificity	0.84	0.84	0.84	0.84	0.84
	F-measure	0.93	0.93	0.93	0.92	0.93
CR ≥ 1 (removing collinearity + COM_3 + FA)	Accuracy	**91.72**	**91.75**	91.27	90.18	**91.80**
	AUC	0.91	0.90	0.92	0.91	0.93
	Sensitivity	0.96	0.97	0.96	0.94	0.97
	Specificity	0.84	0.84	0.84	0.84	0.84
	F-measure	0.93	0.93	0.93	0.92	0.93
CF ≥ 10 (full attributes)	Accuracy	91.44	91.45	91.35	89.90	91.66
	AUC	0.90	0.91	0.92	0.92	0.92
	Sensitivity	0.84	0.85	0.85	0.85	0.85
	Specificity	0.96	0.96	0.96	0.93	0.96
	F-measure	0.89	0.89	0.89	0.87	0.89
CR ≥ 10 (removing collinearity)	Accuracy	**91.55**	**91.57**	90.99	89.67	**91.81**
	AUC	**0.91**	0.91	0.92	0.91	0.93
	Sensitivity	**0.85**	0.85	0.85	0.85	0.85
	Specificity	0.96	0.96	0.95	0.93	**0.97**
	F-measure	0.89	0.89	0.88	0.87	0.89
CR ≥10 (removing collinearity + COM_3)	Accuracy	**91.46**	**91.64**	90.44	89.22	**91.81**
	AUC	0.90	0.91	0.92	0.90	0.93
	Sensitivity	**0.85**	0.85	0.85	0.85	0.85
	Specificity	0.96	0.96	0.94	0.92	**0.97**
	F-measure	0.89	0.89	0.88	0.86	0.89
CR ≥ 10 (removing collinearity + COM_3 + FA	Accuracy	**91.47**	**91.77**	91.36	**91.07**	**91.81**
	AUC	0.90	0.91	0.92	0.92	0.93
	Sensitivity	**0.85**	0.85	0.85	0.85	0.85
	Specificity	0.96	0.96	0.94	0.92	**0.97**
	F-measure	0.89	0.89	0.88	0.86	0.89

Note: The bold numbers denote the metric performance of removed collinear attributes or “removed collinear + COM_3 selected” attributes that showed improvement compared to the full attributes dataset.

In the most valuable CF ≥ 10 dataset, the removal of collinear attributes resulted in several improvements, especially in the accuracy and specificity of the LMT classifier because the LMT is the best RBML classifier across the five metrics. Compared to the CF ≥ 10 (full attributes) dataset, the deleted multicollinear attributes followed by the COM_3 integrated attributes also improved performance. The third stage (deleted multicollinear attributes + COM_3 integrated attributes + FA reduced the attributes that had insufficient cohesion) retained only 10 key attributes, which also improved the two-stage performance. In summary, the proposed three-stage dimension reduction discovered fewer key attributes to enhance the performance of the full-attribute dataset.

**Fig 2:**
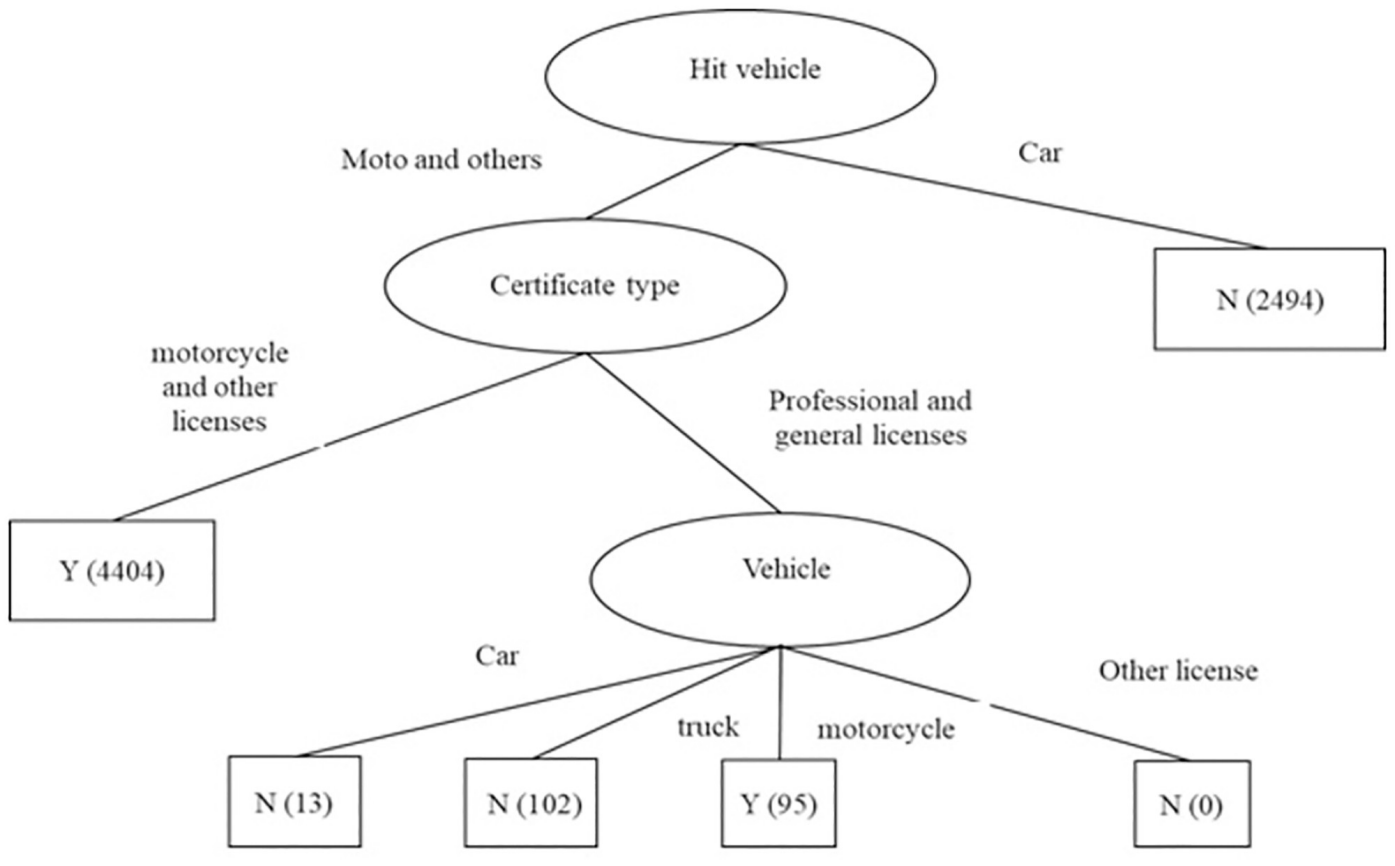
DT diagram of accident severity in the CR ≥ 10 dataset. Note: Y denotes injury or death, N denotes uninjured.

- Generating DT rules. DT is a popular traffic safety method that can quickly interpret complex patterns related to the attributes of road crashes [22]. Further, DT was the first method chosen to understand the rules of road accidents; hence, we used the DT diagram to show the rules of accident severity. The rules of the CR ≥ 10 dataset are shown in Fig 2, which indicates that "hit vehicle" was the most important attribute, and "moto" and "others" were the most prominent causes of injury/death in traffic accidents. The order of the top three attributes was hit vehicle > certificate type > vehicle.

### 4.3 Discussions

Herein, we discuss some key findings from the experimental results.

#### 4.3.1 Key attributes

According to Rolison et al. [43], the causes of vehicle collisions broadly depend on the driver characteristics, including skill level, inexperience, excessive speed, loss of control, failure to detect another vehicle, reckless driving, traffic violations, and drugs and alcohol. Furthermore, road crashes with older drivers often involved making mistakes at intersections, failing to give way when turning, failing to follow signs and signals, not seeing objects, and making incorrect turns and lane changes. Dimension reduction can extract important attributes without reducing the performance of the data analysis, and its purpose is to reduce the number of initial attributes to select a subset that retains sufficient information to obtain satisfactory results. After implementing the experiments, we discovered that the top order of top ten key attributes was Hit_veh > Certificate type > Vehicle > Action_t > Drive_q > Escape > Accident_t > Gender > Job > Trip_p in the most accident frequencies CF ≥10 dataset.

#### 4.3.2 Results of changing the different stages of ordering

In the three-stage dimension reduction, the ordering of the deleted multicollinear attributes and COM_3 integrated attributes would impact their evaluation results, because the two methods considered the target class. Therefore, we experimented with a different ordering of the two combined methods, and the results of deleted multicollinear attributes and COM_3 integrated attributes are listed in Table 10. Furthermore, the results of the COM_3 integrated attributes and deleted multicollinear attributes are listed in Table 11. Comparing Tables 10 and 11 based on the best performance of the LMT classifier, we observe that: (1) the proposed ordering (as Table 10) outperformed the COM_3 integrated attributes, and then deleted multicollinear attributes in the accuracy and AUC of CR ≥ 1 dataset; (2) in the CR ≥ 10 dataset, the proposed ordering is better than the COM_3 integrated attributes and then deleted multi-collinear attributes in accuracy; (3) the FA had no impact on the results of the evaluation metrics of the two datasets; however, it can check the cohesion of those attributes and remove those with insufficient cohesion.

**Table 11:** Results of COM_3 integrated attributes and then removing collinear attributes.

Dataset.	Metric	DT	RIPPER	RF	ET	LMT
CF ≥ 1 (Full attributes)	Accuracy	91.61	91.74	91.54	90.91	91.78
	AUC	0.91	0.90	0.93	0.92	0.93
	Sensitivity	0.96	0.97	0.96	0.96	0.97
	Specificity	0.84	0.84	0.84	0.84	0.84
	F-measure	0.93	0.93	0.93	0.93	0.93
CF ≥ 1 (COM_3)	Accuracy	**91.71**	**91.75**	91.43	90.41	**91.80**
	AUC	0.91	0.90	0.93	0.91	0.93
	Sensitivity	0.96	0.97	0.96	0.95	0.97
	Specificity	0.84	0.84	0.84	0.84	0.84
	F-measure	0.93	0.93	0.93	0.92	0.93
CF ≥ 1 (COM_3 + VIF)	Accuracy	91.60	91.70	91.16	90.01	91.75
	AUC	0.91	0.90	0.92	0.91	0.92
	Sensitivity	0.96	0.97	0.96	0.94	0.97
	Specificity	0.84	0.84	0.84	0.83	0.84
	F-measure	0.93	0.93	0.93	0.92	0.93
CF ≥ 1 (COM_3 + VIF + FA)	Accuracy	91.60	91.70	91.16	90.01	91.75
	AUC	0.91	0.90	0.92	0.91	0.92
	Sensitivity	0.96	0.97	0.96	0.94	0.97
	Specificity	0.84	0.84	0.84	0.83	0.84
	F-measure	0.93	0.93	0.93	0.92	0.93
CF ≥ 10 (Full attributes)	Accuracy	91.44	91.45	91.35	89.90	91.66
	AUC	0.90	0.91	0.92	0.92	0.92
	Sensitivity	0.84	0.85	0.85	0.85	0.85
	Specificity	0.96	0.96	0.96	0.93	0.96
	F-measure	0.89	0.89	0.89	0.87	0.89
CF ≥ 10 (COM_3)	Accuracy	91.58	91.57	91.01	88.25	**91.75**
	AUC	0.91	0.91	**0.93**	0.92	**0.93**
	Sensitivity	0.84	0.85	0.85	0.85	0.85
	Specificity	**0.97**	0.96	0.94	0.90	**0.97**
	F-measure	0.89	0.89	0.88	0.88	0.89
CF ≥10 (COM_3 + VIF)	Accuracy	91.58	91.59	91.41	88.30	**91.78**
	AUC	0.91	0.91	0.92	0.92	**0.93**
	Sensitivity	0.84	0.85	0.85	0.85	0.85
	Specificity	**0.97**	0.96	0.94	0.91	**0.97**
	F-measure	0.89	0.89	0.88	0.86	0.89
CF ≥10 (COM_3 + VIF + FA)	Accuracy	91.58	91.59	91.41	88.30	**91.78**
	AUC	0.91	0.91	0.92	0.92	**0.93**
	Sensitivity	0.84	0.85	0.85	0.85	0.85
	Specificity	**0.97**	0.96	0.94	0.91	**0.97**
	F-measure	0.89	0.89	0.88	0.86	0.89

Note: The bold numbers denote that the metric performance of COM_3 selected attributes/COM_3 selected and removed collinearity attributes has been improved compared to the full attributes dataset.

In summary, the proposed ordering presented the best result, and the FA could rename the factor dimension to obtain the clustering status and intensity of attributes. We used FA to remove the age attribute in the CR ≥ 10 dataset because its absolute loading value was less than 0.5, as shown in Table 8; therefore, the full CR ≥ 10 dataset was reduced from 38 to 10 attributes. That is, FA could find the most useful attributes without reducing the evaluation performance, thereby improving the efficiency of data storage and processing [16].

**Important descriptive statistics.** The following key facts were summarized from the descriptive statistics of the complete CF ≥ 1 dataset, which are listed in Table 12:

- In the vehicle dimension, motorcycles had the largest proportion of road collisions with 61.62% and 56.29% of the vehicle type of victim. The highest percentage of collision types (91.79%) was that of vehicles colliding with vehicles.
- Regarding the human dimension, most road crashes occur at an excessive speed (83.08%), and male drivers are more likely (66.09%) to be involved in collisions. Furthermore, the accident cause is the driver at 66.19%, which is the same as the cause of vehicle collisions broadly depending on the characteristics of the drivers [43].
- The road class, separating fast and slow lanes, road type, accident location, and signal type had the highest percentage of collision types in the road dimension.

**Table 12:** Road accident factors and attribute value more than 50%.

Dimension	Attribute	Value (%)	
Vehicle	Vehicle	Motorcycles (61.62%)	Others (38.38%)
	Vehicle type of victim	Motorcycles (56.29%)	Others (43.71%)
	Hit vehicle	Vehicles colliding with vehicles (91.79%)	Others (8.21%)
Human	Overspeed	Yes (83.08%)	No (16.92%)
	Gender	Male drivers (66.09%)	Female (33.91%)
	Accident cause	Drivers (66.19%)	Others (33.91%)
Road	Road class	Urban road (76.98%)	Others (23.02%)
	Separating fast and slow lanes	No fast and slow lane separation (80.94%)	Others (19.06%)
	Road type	Intersection road (60.59%)	Others (39.41%)
	Accident location	Intersection road (58.00%)	Others (42.00%)
	Signal type	No (58.49%)	Others (41.51%)

**Some suggestions.** Based on discussions (1)–(3) and the results of this study, we provide the following suggestions to the government and stakeholders for reference:

- It is necessary for the government to formulate basic laws and regulations for extensive traffic management and inform road users of the rules, methods, and essentials of safe road use. The government must manage, publicize, supervise, and ban them from achieving their results.
- Traffic-related units must implement safe driving training and education (laws and regulations, safe driving, and accident prevention).
- A rigorous test must be required to obtain a driver’s license to determine the drivers who can safely use a road.
- Law enforcement units must continue to implement rigorous management and supervision, and ban offenders to eliminate drivers’ mentality of luck and speculation.
- Road management agencies must make proper corrections and improve the quality of road projects to maintain driving safety, and the relevant driving safety settings must be fully applied.
- Regarding the regulations, methods, and matters that should focus on pedestrians and slow vehicles, law enforcement agencies should be responsible for publicizing and educating them to prevent them from being affected or implicated in the safety of vehicles owing to illegal road use.

## 5. Conclusion

This study proposed an RBML classifier based on accident frequencies and a three-stage dimension reduction to explore the factors of road accident severity. After verifying the experimental results and discussions, it was evident that the ordering of the removed collinear attributes + COM_3 integrated attributes obtained a better result, and the FA could remove the attributes that lacked cohesion and rename the factor dimensions. Overall, this study contributes to the literature by exploring the factors of road accident severity as follows:

- We proposed a three-stage dimension reduction (including deleting multi-collinear, integrating four attribute selection algorithms, and FA reducing attributes) to find the key attributes, and the top 10 order of key attributes was Hit_veh > Certificate type > Vehicle > Action_t > Drive_q > Escape > Accident_t > Gender > Job > Trip_p in the most accident frequencies CF ≥ 10 dataset.
- Five RBML classifiers were applied to classify the injury severity of road accidents and generate rules for road accident severity. The results showed that the LMT classifier performed the best because it uses the LogitBoost algorithm [30] to gradually improve the logistic regression model, and the CART algorithm [31] to prune the tree.
- The primary factor of road crashes was the driver, at 66.19%, which is the same as the causes of vehicle collisions, which broadly depend on the characteristics of the drivers [43].
- Six suggestions were also provided to the government and stakeholders for reference.
- In future work, we will collect more detailed data, such as focusing on younger drivers, motorcycles, and gender of drivers, to find significant factors. Furthermore, text mining can be used to build a topic model and classify injury severity.

## Supporting information

S1 Data (CSV)
